# Exclusive Breastfeeding and its associated factors among mothers of children aged 6 to 23 months in Biratnagar Metropolitan City, Nepal

**DOI:** 10.1371/journal.pone.0356380

**Published:** 2026-08-18

**Authors:** Kritika Roka, Paras Modi Pangeni, Manish Rajbanshi, Susmita Thapa, Aashish Rana, Shakuntala Chapagain

**Affiliations:** 1 Department of Public Health and Community Medicine, Chitwan Medical College, Tribhuvan University, Chitwan, Nepal; 2 Central Department of Public Health, Institute of Medicine, Tribhuvan University, Kathmandu, Nepal; Mizan-Tepi University, ETHIOPIA

## Abstract

Exclusive breastfeeding (EBF) is one of the most vital interventions to promote child’s growth, survival and maternal health, yet the rate of EBF remains suboptimal in many developing nations, leading to increase in infant and childhood mortality and morbidity. This study aimed to assess the practice of exclusive breastfeeding and its associated factors among mothers of children aged 6–23 months in Biratnagar Metropolitan City, Nepal. A cross-sectional study was conducted among 202 mothers with children aged 6–23 months in Biratnagar, Nepal, employing a Population Proportionate to Size (PPS) technique for sampling. The participants were asked about their feeding practice to infants since birth with a face-to-face interview using a structured questionnaire. Written consent was taken for each participant prior to the interview. Among 202 mothers (Mean ± S.D = 25.65 ± 4.15), nearly one-fourth of the participants reported practicing early breastfeeding. Most of the participants consumed plain water with milk (53.5%), followed by infant formula milk (35.6%) in early infancy. One fourth of the participants (21.8%) had adequate knowledge of EBF. The reported exclusive breastfeeding was 37.6% with the median duration of four months. The major reason for mixed feeding was mother's perceived milk as not sufficient, followed by work. In multivariable analysis, mothers from joint/extended families (AOR = 1.95, 95% CI: 1.23–4.09), illiterate mothers (AOR = 2.90, 95% CI: 1.14–7.41), and mothers without breastfeeding problems (AOR = 8.23, 95% CI: 2.97–22.74) were significantly more likely to practice EBF. Despite the national and local efforts on nutrition, our study revealed the exclusive breastfeeding rate was low, with lower participants having adequate knowledge on EBF. This implies the need for counselling mothers regarding EBF on ANC, PNC visits. As the breastfeeding problem was significantly hindering breastfeeding practice, counselling on interventions to deal with breastfeeding problems would benefit the improvement in practice.

## Introduction

Exclusive breastfeeding (EBF), defined as feeding infants only breast milk for the first six months of life is fundamental to child survival, healthy growth, and development [1]. Breast milk provides essential nutrients and acts as the infant's first immunization, offering protection against major childhood illnesses including diarrhea, respiratory infections, and malnutrition, which remain leading causes of infant and neonatal mortality globally [1,2]. EBF is beneficial for the health and well-being of both child as well as mother. Mothers practicing EBF have significantly lower risks of breast and ovarian cancers along with metabolic diseases like diabetes and blood pressure [3].

Conversely, early introduction of complementary foods or suboptimal breastfeeding increases mortality and disease burden among children under five, and is associated with greater likelihood of chronic diseases such as obesity and diabetes in early adulthood [4,5]. EBF is estimated to prevent more than 820,000 global deaths of under-five (U-5) children annually [6]. Despite its evidence-based benefits, EBF rates remain suboptimal worldwide. Between 2017–2023, only 48% of infants were exclusively breastfed worldwide with regionally varying rates of EBF from 26% to 60% in North America and South Asia, respectively [7]. To improve the current scenario and prevent childhood mortality rate, the World Health Organization (WHO) aims to achieve at least 50% EBF globally by 2025 and 70% by 2030 [2].

In World Health Assembly (WHA) 2022, EBF was recognized as a powerful and cost-effective intervention to reduce the lifelong burden of non-communicable diseases and it also emphasized on reinforcing the International Code of Marketing of Breast Milk Substitute (BMS)**,** for discouraging harmful marketing practices [8]. Nepal has aligned with these intenational commitments through several legislative and programmatic efforts. In 1992, Nepal enacted the BMS (Control of Sale and Distribution) Act for regulating the marketing of breast-milk substitute and promoting EBF [9]. The Multi Sectoral Nutritional Plan III (MSNP-III) targets to achieve ≥  90% EBF among infants by 2030 [10]. Baby Friendly and Hospital Initiative (BFHI) was implemented in 1999 for promotion of EBF [10]. Community based comprehensive nutrition programs like Infant and Young Child Feeding (IYCF) and Suaahara program are focused on improving nutritional status through emphasis on EBF and other nutritional interventions [11,12]. Despite such efforts, Nepal Demographic and Health Survey (NDHS) report found a sharp decrease in the EBF rate from 66% to 56% in 2016 and 2022, respectively [13].

The practice of EBF in Nepal varies across provinces with Karnali and Sudurpaschim province having the highest rates (73.8% each). Also there is a disparity in EBF practice between rural and urban settings. For example, Sudurpaschim Province (rural: 81.1% vs urban: 69.8%) and Bagmati province (67.3% vs 37.8%) [13].

This highlights the need to emphasize interventions to promote EBF practice in urban areas. Along with the place of residence (urban or rural), various demographic and maternal factors like age of mother, education and occupation are linked with the feeding practices [4,14]. Studies have also identified marketing of infant milk products, antenatal and postnatal counseling and husband support influencing the EBF rates [2,4,5,11,15]. The varying EBF practice is shaped by socio-cultural factors including sex of the child and prelacteal feeding practices, especially in culturally influenced nation like Nepal [16]. Studies in rural areas of mid-western and central Nepal indicate lower practice of EBF, despite adequate maternal knowledge [11,17,18]. However, limited evidence exists regarding EBF practice in urban settings of eastern Nepal.

Biratnagar, being the capital of Koshi Province and one of the largest urban centers in eastern Nepal, serves as a major economic and cultural hub. In such urban settings, factors such as increased exposure to media, rapid urbanization, aggressive marketing of breast milk substitutes, and the availability of artificial milk may act as barriers to EBF [19]. Therefore, identifying the determinants of infant feeding practices particularly in urban areas of Koshi Province is essential for informing policymakers and program implementers about these barriers and for promoting EBF through targeted and strategic interventions. Thus, this study aimed to assess the practice of exclusive breastfeeding and its associated factors among mothers of children aged 6–23 months in an urban setting of Biratnagar Metropolitian City, Nepal.

## Materials and methodology

### Study design and setting

A community-based cross-sectional study was conducted among mothers of children aged 6–23 months residing in Biratnagar Metropolitan City (BMC). Administratively, the city has a total of 19 wards and represents about 19.7% of the total population of Morang District. According to the 2021 National Census [20], BMC has a total population of 243,927, including 121,954 males and 121,973 females. According to BMC Health Division, a total of 5,310 mothers with children aged 6–23 months and 12858 U-5 children were residing in the city at the time of the study.

### Study population

The study was carried out among mothers of children aged 6–23 months who had been residing in BMC for at least one year prior to data collection.

Inclusion criteria: The mother having children aged between 6 months and under 2 years at the time of data collection and had provided informed consent to participate in the study.

Exclusion criteria: If mothers were not available during the first home visit, a second follow-up visit was conducted. Those who remained unavailable during the second visit were excluded from the study, and the next eligible mother residing in the nearest household was selected for participation. Similarly, mothers with hearing or speech impairments that hindered effective communication during the interview process were also excluded.

### Sample size and sampling technique

The sample size for this study was determined using Cochran’s formula [21]:

n_0_=Z^2^pq /d^2^

n_0_ ≈ 210

The initial sample size was (n_0_ ≈ 210).

**Where:**

Z= 1.96 (standard normal deviate at 95% Confidence Interval)

p= 0.163 (estimated proportion of EBF was taken from a similar study) [12]

q= 1−p =0.837

d= 0.05 (allowable error)

N= 5310 (total number of eligible participants inBMC)

The final sample size (n) was calculated using a finite population formula [21],

n=1+N (n_0_−1)/n_0_

n ≈ 202

The minimum required sample size for this study was 202.

A multistage sampling technique was used to select the required number of participants. Among the 19 wards of BMC, 6 wards (Wards 2, 7, 8, 13, 17, and 18) were selected through simple random sampling using the lottery method. Subsequently, the number of participants from each selected ward was determined using the PPS method. The required number of sample for each selected ward was: ward 2 = 28, ward 7 = 22, ward 8 = 61, ward 13 = 26, ward 17 = 32, and ward 18 = 33. Then, the list of all eligible participants was obtained from the Health Division of BMC. The required number of participants from the selected wards were chosen using a lottery method via computer generated numbers. The house of selected participants was identified with the help of Female Community Healthcare Volunteers (FCHVs) of respective wards. To minimize non-response bias, if a selected participant was unavailable during data collection, the alternative participant was approached as a replacement (S2 File).

### Tools and measures

This study adopted tool from the WHO and UNICEF guidelines, with modifications based on extensive literature review [8,14]. Nepali translated language tool was used for the data collection. The tool was pretested among 10% of the sample in a similar population in non-selected ward of BMC. The necessary editing and wording was done after the pretesting. The Cronbach's alpha coefficient (α = 0.83) was used to determine and measure internal consistency and reliability (α = 0.83).

The tool included 32 items covering EBF practices and associated factors- socio-demographic, obstetric, health-related, and knowledge-based. The questionnaire was organized into four sections. The first section included socio-demographic factors that could affect breastfeeding practices like mother’s age, religion, ethnicity, family type, education and occupation, both maternal/paternal education and occupation, child’s birth weight and gender. The second section consisted of questions related to knowledge of EBF, determined by six topics: meaning, time of initiation, first feeding, benefits to mother and child, and management of breastfeeding when child is ill. The third section focused on Obstetrics and Health service-related factors, comprising topics such as parity, place and mode of delivery, Antenatal Care (ANC) visits (including frequency), Postnatal Care (PNC) visits (including frequency), counselling of EBF received from FCHVs or during the ANC or PNC visits, and breastfeeding related problems experienced within the first six months after delivery. The last section comprised questions regarding the EBF practices such as: initiation time of breastfeeding, feeding practices within the first six months, duration of EBF, and reasons for not continuing EBF.

### Operational definition

#### Reported EBF practice.

It is an infant being fed only breast milk as the sole source of nutrition for the first six months of life [2]. No other liquids, solids, or water is consumed by the infants, except vitamins, minerals, and medicines. In this study, EBF was assessed retrospectively based on maternal recall among mothers of children aged 6–23 months. Mothers were asked a series of questions regarding the introduction of specific foods and liquids, including plain water, infant formula, animal milk, juice, clear broth, yogurt, thin porridge, honey, and any other food or liquid (except medicines, vitamins, and ORS) during the first six months of life. If the child had received any of these items during the first six months, the practice was classified as non-exclusive breastfeeding (non-EBF).

#### Knowledge of EBF.

It was classified based on Bloomberg's taxonomy which considered 80% of the total score as a cutoff point [22]. Therefore, the adequate knowledge in our study was a score of 4 or greater which is 80% of 6. Finally, the inadequate level of knowledge was less than 4.

### Data collection

The data collection was carried out between 3^rd^ October 2023–3^rd^ January 2024. Face-to-face interviews were conducted using structured questionnaires. The Principal Investigator (KR) was responsible for data collection. The FCHVs helped to identify the households of mothers, and assisted during the interview. Each interview took around 15–20 minutes to complete.

### Data management and analysis

Data were systematically entered, cleaned, coded, and validated using EpiData, and subsequently analyzed in International Business Machines-Stastical Package for the Social Sciences (IBM-SPSS) Statistics. Descriptive statistics including frequencies, percentages, means, and standard deviations were used to summarize participants’ socio-demographic and economic characteristics. Inferential analysis was conducted using the chi-square test and bivariate binary logistic regression to assess associations between individual variables and EBF. Multiple regression models were developed using varying inclusion thresholds (p-values <0.1, < 0.2, and <0.3) and compared with a theoretically informed full model. Variables meeting the p-value threshold of <0.3 were then included in the final multivariable logistic regression model to adjust for potential confounders. Model fitness was evaluated using the Hosmer–Lemeshow goodness-of-fit test (p < 0.05) and the Nagelkerke R² statistic (R² = 0.25). Statistical significance was determined at p < 0.05. Adjusted odds ratios (AORs) with 95% CI were reported to quantify the strength and precision of associations.

### Ethical consideration

Ethical approval was taken from Institutional Review Committee of Chitwan Medical College, Tribhuvan University (CMC-IRC-080/081–098). A letter of support was obtained from Biratnagar Metropolitan City’s office for data collection. Written consent form was obtained from the participants. Anonymity was maintained throughout the study to protect participants’ privacy, and they were granted the right to withdraw from the study at any time.

## Result

### Socio-demographic characteristics of the participants

A total of 202 mothers participated in this study with ages ranging from 19 to 40 years (Mean ± S.D = 25.65 ± 4.15). Among them, 87.6% of them were Hindu, 66.8% were living in joint family. Majority of them had primary education (40.7%). About three-fourths of the participants were involved as homemaker (Table 1).

**Table 1:** Socio-demographic characteristics of the participants.

Characteristics	Number (n)	Percentage (%)
**Age (years)**		
Young adult (> 18 –30)	180	89.1
Middle-aged adults (31 –45)	22	10.9
Mean ± S.D (25.65 ± 4.15)		
**Religion**		
Hindu	177	87.6
Non-Hindu	25	12.4
**Ethnicity**		
Brahmin	31	15.2
Chhetri	21	10.3
Janajati	55	27.2
Madhesi	62	30.6
Muslim	25	12.3
Dalit	9	4.4
**Family type**		
Joint/ Extended	135	66.8
Nuclear	67	33.2
**Educational Status of mother**		
Illiterate	37	18.3
Can only Read and Write	9	4.5
Primary	67	33.2
Secondary	57	28.2
Bachelor’s and above	32	15.8
**Educational Status of husband**		
Illiterate	34	16.8
Can only Read and Write	6	2.9
Primary	62	30.7
Secondary	67	33.3
Bachelor’s and above	33	16.3
**Main occupation of mother**		
Homemaker	142	70.3
Service	23	11.4
Business	18	8.9
Daily wages	12	5.9
Other (Agriculture, Foreign employment)	7	3.5
**Main occupation of husband**		
Daily wages	65	32.2
Business	64	31.7
Service	40	19.8
Agriculture	14	6.9
Foreign employment	11	5.4
Homemaker	8	4
**Age of child (in months)** **Range**	*6-23*	
6 −12	89	44.1
13 - 17	55	27.2
18 −23	58	28.7
**Sex of child**		
Male	106	52.5
Female	96	47.5
**Birth weight of child**		
Low-birth weight (<2.5 kg)	39	19.3
Normal (≥2.5 kg)	163	80.7

### EBF related characteristics of the participants

Table 2 shows only about one-fourth (27.2%) accurately identified EBF as providing only breast milk (with medicines if required) for the first six months, whereas the majority held misconceptions. More than half of the mothers (56.4%) knew that breastfeeding should be initiated within the first hour after delivery, and almost all (97.5%) correctly identified breast milk as the appropriate first feed. Regarding the benefits of EBF, mothers most commonly recognized its role in preventing maternal anemia and supporting the child’s growth and development. Knowledge on breastfeeding during infant illness was strong, with 95% reporting that breastfeeding should be continued when the child is sick. Overall, the responses showed good breastfeeding knowledge but a notable gap existed about the correct definition of EBF.

**Table 2:** Knowledge regarding exclusive breastfeeding among mothers.

Characteristics	Number (n)	Percentage (%)
**Information on the meaning of EBF**		
Providing Breast milk and water for up to 3 months	63	31.2
Providing only breast milk and also allow to receive medicine (if necessary) to children up to 6 months	55	27.2
Providing breast milk and honey to child up to 1 year	24	11.9
Providing only breast milk to child up to 2 years	60	29.7
**Initiation of breastfeeding after delivery**		
Within an hour	114	56.4
After an hour	81	40.1
After 2 hours	6	3.0
After long time	1	0.5
**First feeding after delivery**		
breastmilk	197	97.5
water	3	1.5
Others (Honey, formula milk)	2	1.0
**Benefits of EBF to mother**		
Prevent from anemia	74	36.6
Protect against allergies	62	30.7
Natural family planning	41	20.3
Increase appetite	25	12.4
**Benefit of** **EBF for child**		
Promote growth and development	129	63.8
Protect from cold	70	34.7
Early eruption of teeth	3	1.5
**Practice breastfeeding when the infant is ill**		
Continue breastfeeding	192	95.0
Stop breastfeeding till child recovery	8	4.0
Stop breastfeeding for 1 day	2	1.0

### Level of knowledge

**Fig 1:**
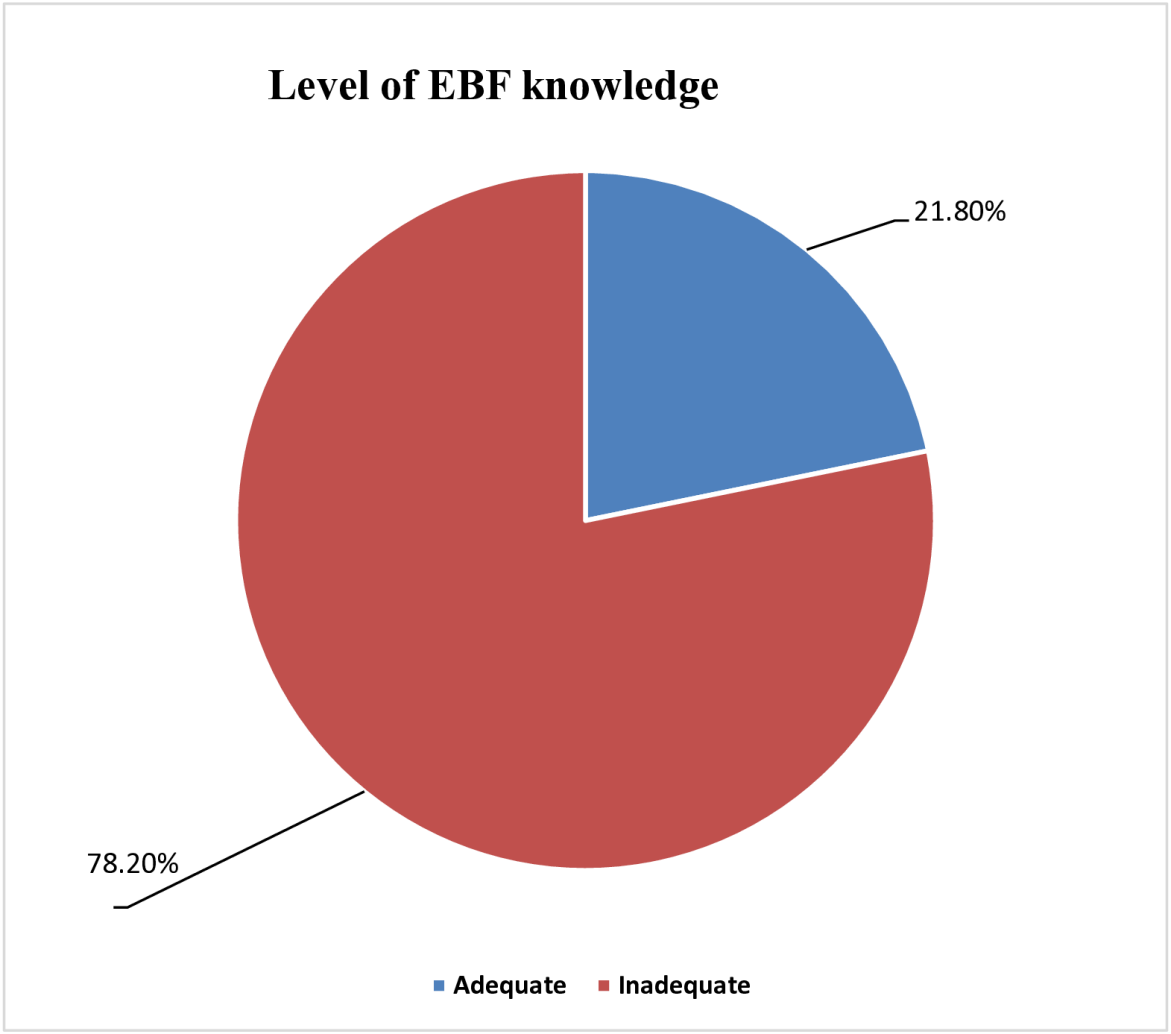
Level of EBF knowledge.

As shown in Fig 1, only 21.8% of the participants had an adequate knowledge on EBF practice.

Table 3 indicates that more than half of the women (56.9%) had experienced more than one birth, and almost all (97%) had delivered in a health facility. The majority (92.6%) reported attending at least four ANC visits, while comparatively fewer (23.7%) had completed more than three PNC visits. About two-thirds of the participants had received breastfeeding counseling. More than one-fourth (27.2%) reported facing breastfeeding-related problems, with low milk production identified as the most common issue.

**Table 3:** Health related characteristics of exclusive breastfeeding practice.

Characteristics	Number (n)	Percentage (%)
**Parity**		
Primiparous	87	43.1
Multiparous	115	56.9
**Place of delivery**		
Institution	196	97.0
Home	6	3.0
**Mode of delivery**		
Normal	107	53.0
CS	95	47.0
**ANC visit**		
Yes	187	92.6
No	15	7.4
**Number of ANC (n = 187)**		
<4 times	13	6.9
≥4 times	174	93.1
**PNC visit**		
Yes	197	97.5
No	5	2.5
**Number of PNC visits (n = 197)**		
<3 times	151	76.6
≥3 times	46	23.4
**EBF counseling**		
Yes	119	58.9
No	83	41.1
**Breastfeeding problems from birth of child till 6 months**		
Yes	55	27.2
No	147	72.8
If Yes, Problems * (n = 55)*denotes multiple response		
Low milk production	39	70.9
Painful breasts, cracked nipples, or inverted nipples	19	34.5
Difficult in sucking	8	14.5

### Exclusive breastfeeding practice and its hindering factors

Table 4 shows that a majority of mothers (71.8%) initiated breastfeeding only after one hour of birth. More than half of the infants (62.4%) were introduced to other foods before six months, with water being the most commonly reported (53.5%), followed by infant formula milk (35.6%) and honey (24.8%).

**Table 4:** Patterns of breastfeeding initiation, mixed feeding practices, and exclusive breastfeeding status.

Characteristics	Number (n)	Percentage (%)
**Practice of breastfeeding initiation**		
< 1 hour	57	28.2
≥ 1 hour	145	71.8
Consumption from birth till 6 months**denotes multiple response		
Plain water	108	53.5
Infant formula milk	72	35.6
Animal milk	47	23.3
Fruit juice	17	8.4
Soup	20	9.9
Yogurt	12	5.9
Thin porridge	27	13.4
Honey	50	24.8
Other liquids except medicine	16	7.9
**Reported Exclusive Breastfeeding Practices**		
Yes	76	37.6
No	126	62.4
*Median (Inter-quartile range) =4(3), Min/Max = 0/7*		
**If not, reasons (n = 126)**		
Mother’s milk is not sufficient	66	52.4
Work /cultural practices	38	30.2
Thought that infant is thirsty	20	15.8
Family pressure to feed other things	2	1.6

Less than two in five mothers (37.6%) reported the practice of exclusive breastfeeding, whereas a comparatively larger proportion (62.4%) did not exclusively breastfeed their infants. The median duration of exclusive breastfeeding was 4 months (IQR: 3 months), with reported durations ranging from 0 to 7 months. Among mothers who did not practice exclusive breastfeeding, the perception of insufficient breast milk was by far the most frequently cited reason (52.4%). Work-related or cultural practices were the next most common reason (30.2%), followed by the belief that the infant was thirsty (15.9%). Only a very small proportion (1.6%) attributed early introduction of other feeds due to family pressure.

### Factors associated with EBF practice

The bivariate analysis in the (S3 File) shows that mothers from joint/extended families were twice more likely to practice EBF compared to those from nuclear families (COR = 2.28, 95% CI: 1.19–4.36). Maternal and paternal educational status also showed significant associations. Illiterate mothers were 3.47 times more likely to practice EBF than literate mothers (COR = 3.47, 95% CI: 1.65–7.27). Similarly, illiterate fathers had twice higher odds of their spouses practicing EBF (COR = 2.13, 95% CI: 1.01–4.49). Mothers who visited PNC three or more times were less likely to practice EBF compared to those with fewer than three PNC visits (COR = 0.44, 95% CI: 0.21–0.95). Breastfeeding-related difficulties displayed the strongest association; mothers reporting no breastfeeding problems were more than seven times more likely to practice EBF than those who had breastfeeding problems (COR = 7.42, 95% CI: 2.99–18.39). Level of knowledge, parity, mode of delivery, and other sociodemographic variables were not significantly associated with EBF practice.

### Factors associated with EBF practice

In Table 5 mothers residing in joint/extended families had almost twice higher odds of the EBF compared to those living in nuclear families (AOR: 1.95, 95% CI: 1.23–4.09). Similarly, mothers who were illiterate were almost three times more likely to practice EBF than literate mothers (AOR: 2.90, 95% CI: 1.14–7.41). Breastfeeding problems showed the strongest association with the EBF. Mothers who reported no breastfeeding problems were eight times more likely to have EBF practice compared to those who reported breastfeeding difficulties (AOR: 8.23, 95% CI: 2.97–22.74).

**Table 5:** Multivariate logistic regression analysis of factors associated with EBF practice.

Characteristics	COR 95% CI	p-value	AOR 95% CI	p-value
**Family Type**				
Nuclear (1)	Ref			
Joint/Extended	2.28 (1.19-4.36)	0.012*	1.95 (1.23-4.09)	0.047*
**Educational Status of mother**				
Literate (1)	Ref			
Illiterate (2)	3.47 (1.65-7.27)	0.001*	2.90 (1.14-7.41)	0.025*
**Educational Status of father**				
Literate (1)	Ref			
Illiterate (2)	2.13 (1.01-4.49)	0.046*	1.10 (0.42-2.90)	0.836
**Husband’s Occupation**				
Agriculture (1)	Ref			
Non-agriculture (2)	2.32 (0.62-8.62)	0.206	4.07 (0.93-17.67)	0.061
**Level of Knowledge**				
Adequate (1)	Ref			
Inadequate (2)	1.58 (0.76-3.25)	0.213	0.96 (0.40-2.27)	0.928
**Parity**				
Primiparous (1)	Ref			
Multiparous (2)	1.51 (0.84-2.70)	0.166	1.22 (0.62-2.40)	0.559
**Number of PNC visit**				
<3 times (1)				
≥3 times (2)	0.45(0.21-0.95)	0.038*	0.53 (0.23-1.23)	0.144
**Breastfeeding problems**				
Yes (1)	Ref			
No (2)	7.42(2.99-18.39)	<0.001*	8.23(2.97-22.74)	<0.001*

## Discussion

The EBF prevalence in this study (37.6%) was lower than the national (56.0%) and Koshi Province (52.7%) estimates reported by the NDHS 2022 [13]. The lower prevalence may be attributable to Biratnagar's rapidly urbanizing environment, where structural and socioeconomic factors including increased maternal employment, early return to work, inadequate maternity and workplace breastfeeding support, greater exposure to breast milk substitute marketing, and reduced family support may hinder optimal exclusive breastfeeding practices [10,12,17,23,24].The finding was comparable to the study conducted in Saptari (43.6%) [25] and Lalitpur (43.3%) [26], suggesting that urbanization and related socioeconomic conditions may influence breastfeeding practices. Despite Nepal’s national policies and strategies promoting optimal IYCF [22,24], the findings indicate suboptimal translation of these provisions into practice, particularly in urban and semi-urban settings [24,27]. Consequently, rapidly urbanizing cities such as Biratnagar require comprehensive structural and policy-level interventions in addition to conventional awareness and education programs.

The EBF was lower than estimates from South Asia (61%), India (65%), and Pakistan (48%) [7,28,29] and substantially lower than rates reported in rural areas of India and Ethiopia [30,31]. Higher EBF rates in rural settings may reflect stronger traditional practices, lower maternal workforce participation, and greater community support [16–18].

The lower EBF prevalence was consistent with studies conducted in Australia, urban Nevada, and the United States [32–35]. Evidence from high-income settings also demonstrates declining EBF continuation after the early postpartum period, consistent with the short median EBF duration observed in this study [36]. These findings highlight the influence of urbanization and structural factors, including maternal employment, inadequate workplace support, limited maternity leave, and insufficient breastfeeding-friendly environments [37–43]. Therefore, rapidly urbanizing settings such as BMC require structural and policy-level interventions alongside awareness-based approaches.

The present study found that only 21.8% of mothers had adequate knowledge of BF, highlighting a considerable knowledge gap. This finding is comparable to those reported in Bangladesh (34.5%) [44] and Yemen (24.0%) [45], but substantially lower than estimates from India (69.8%) [46], Nigeria (76.0%) [47], and Kenya (66.6%) [48]. The observed differences may reflect variations in maternal educational attainment, the quality and accessibility of maternal and child health services, and the coverage of breastfeeding promotion programs across settings. In the present context, inadequate maternal education, insufficient antenatal and postnatal breastfeeding counseling, and limited community-based health education initiatives may have contributed to the low level of EBF knowledge among mothers [13,16,17,24,49].

Only 28.2% of mothers started early initiation of breastfeeding. This finding is comparable to previous evidence from Nepal (19.7%) [50]. However, it remains lower than other studies conducted in Nepal (41%–66.4%) [51–53], South Asia (41.0%) [52] and the systematic review of LMICs (49.0%) [54]. The low prevalence of early initiation of breastfeeding observed in this study may be attributed to several factors, including higher rates of cesarean delivery, prelacteal feeding practices, inadequate counseling during the antenatal and immediate postnatal periods, and sociocultural beliefs that discourage colostrum feeding [16,49,53,55–57]. Furthermore, suboptimal implementation of the BFHI and limited access to skilled breastfeeding support during delivery and the immediate postpartum period may have further contributed to delayed initiation of breastfeeding [58,59].

Illiterate mothers demonstrated nearly three times higher odds of practicing EBF compared to literate mothers. Similar evidences were generated from study conducted in Nepal (NDHS 2022) [13], China [60] and Ghana [57]. This finding is also supported by evidence from a Nepal based comparative study which shows huge difference in EBF practice between employed (13.8%) and unemployed mothers (81.2%) [58]. The lower practice of EBF among educated mothers may reflect competing occupational and lifestyle demands that make sustained exclusive breastfeeding more difficult, particularly in urban settings where balancing employment and infant feeding is often challenging [13,27,58,61]. Additionally, greater exposure to breastmilk substitute marketing through healthcare systems, media, and commercial channels may further influence feeding choices among educated mothers [62,63]. Conversely, in resource-constrained settings, traditional norms and limited access to breastmilk substitutes may unintentionally promote adherence to EBF practices [57,63,64].

Breastfeeding-related challenges, including perceived low milk production and physical difficulties such as breast pain and engorgement, were significantly associated with suboptimal EBF practices [18]. Mothers without such difficulties were more likely to practice EBF [65–67]. Similar associations have been reported in studies from Nepal [67,68], India [69], Bangladesh [70], Srilanka [71], and Vietnam [72], as well as in global systematic reviews identifying perceived milk insufficiency as an underlying reason of early discontinuation, even when there is no actual physiological problem [66]. However, evidence from countries such as Ethiopia and Sri Lanka indicates that high EBF practice can be sustained despite breastfeeding challenges when supported by strong community networks, effective counseling services, and skilled health worker support [71]. This suggests that breastfeeding difficulties alone do not determine feeding outcomes but are mediated by contextual factors such as maternal confidence, family support, and access to appropriate guidance [4,24,52,54,67].

Mothers from joint families had nearly twice the odds of practicing EBF compared with those from nuclear families. This may be attributed to enhanced emotional, practical, and informational support from extended family members [4,10,16]. Similar findings have been reported in several studies from Nepal [10,16,24], as well as in India and Bangladesh [44,69,70], where support from extended family members may help mothers continue EBF by sharing household responsibilities and providing emotional encouragement.

Overall, the findings highlight that perceived breastmilk insufficiency is often a psychosocial rather than physiological phenomenon, influenced by inadequate knowledge, insufficient postnatal counseling, and cultural misconceptions [16,29,66,72]. Similarly, breastfeeding-related physical problems are frequently linked to modifiable factors such as improper positioning and latching, which can be effectively addressed through timely lactation support [10,61,72].

## Conclusion

The EBF rate in Biratnagar is comparatively lower than the national EBF rate, which highlights the need to shift focus towards the urban settings for improvement. Factors like maternal education, breastfeeding problems emerged as the potential hindrance in EBF, implying the influence of both individual and structural factors on feeding practices. The high reliance on infant formula milk and low knowledge level further reflects the missed opportunity for adequate counselling on EBF.

These findings indicate the need to strengthen the interventions targeted in urban areas, especially supporting working mothers through safer breastfeeding workspace. Additionally, it emphasizes the importance of effective counseling regularly during ANC, PNC visits to address breastfeeding problems like perceived insufficient breast milk which was a major concern identified in this study.

### Strength and limitation

This study provides valuable evidence on EBF practices from a rapidly urbanizing city in Koshi Province, where limited data are available. The inclusion of children aged 6–23 months enabled a comprehensive assessment of feeding practices across the first six months of life, while the use of structured questionnaires and standardized procedures enhanced data reliability. However, several limitations should be considered. The reliance on maternal recall may have introduced recall bias, particularly for older infants, and the use of recall since birth rather than the WHO-recommended 24-hour recall limits direct comparability with national and global estimates. The measurement of maternal knowledge using a limited number of items and dichotomization based on Bloom’s cut-off may have reduced sensitivity to detect meaningful variations, potentially explaining the lack of observed association with EBF practices.

### Implications of the study

To enable an optimal EBF rate in Nepal, policies driven by structural and local evidence are essential. Therefore, further work should be targeted towards urban settings like Biratnagar, employing a prospective study design to minimize recall bias. Furthermore, qualitative methodologies like focus group discussion, in-depth interviews may possibly provide deeper insights into perceived breastmilk insufficiency, knowledge gaps, and the reasons for early introduction of infant milk. Finally, studies should aim to understand the quality of counselling sessions received by the participants as this study merely gained information on whether they attended ANC, PNC and the frequency of the visits, without the adequacy of the information provided.

## Supporting information

S1 File Dataset. (XLSX)

S2 File Table 1. (DOCX)

S3 File Table 6. (DOCX)
