# Supplementary material for: Exclusive Breastfeeding and its associated factors among mothers of children aged 6 to 23 months in Biratnagar Metropolitan City, Nepal
Source: PLoS One. 2026 Aug 18;21(8):e0356380. doi: 10.1371/journal.pone.0356380 (PMC13484968; doi:10.1371/journal.pone.0356380)
Supplement: Supplementary file 1 [file pone.0356380.s002.docx]

**Table 1: Sampling distribution of selected wards**

| **Ward** | **Eligible Population** | **PPS** | **Ward** | **Eligible Population** | **PPS** |
| --- | --- | --- | --- | --- | --- |
| 1 | 313 |  | 11 | 282 |  |
| 2 | 181 | 28 | 12 | 411 |  |
| 3 | 456 |  | 13 | 166 | 26 |
| 4 | 480 |  | 14 | 299 |  |
| 5 | 383 |  | 15 | 274 |  |
| 6 | 432 |  | 16 | 150 |  |
| 7 | 143 | 22 | 17 | 204 | 32 |
| 8 | 391 | 61 | 18 | 211 | 33 |
| 9 | 126 |  | 19 | 160 |  |
| 10 | 245 |  |  |  |  |
| **Total** | **5310** |  |  |  | **202** |

PPS: Population Proportionate to Size
