# Supplementary material for: Exclusive Breastfeeding and its associated factors among mothers of children aged 6 to 23 months in Biratnagar Metropolitan City, Nepal
Source: PLoS One. 2026 Aug 18;21(8):e0356380. doi: 10.1371/journal.pone.0356380 (PMC13484968; doi:10.1371/journal.pone.0356380)
Supplement: Supplementary file 2 [file pone.0356380.s003.docx]

**Table 6: Bivariate analysis of factors associated with EBF practice**

| **Characteristics** | **Exclusive Breastfeeding** | | **COR**  **(95% CI)** | **P-value** |
| --- | --- | --- | --- | --- |
|  | **Yes (%)** | **No (%)** |  |  |
| **Age group of mothers in years** |  |  |  |  |
| Young Adult (18-30) | 67 (37.2) | 113 (62.8) | Ref |  |
| Middle aged Adult (31-45) | 9 (41.9) | 13 (59.1) | 1.16(0.47-2.87) | 0.736 |
| **Religion** |  |  |  |  |
| Muslim | 11(44) | 14 (56) | Ref |  |
| Hindu | 65(36.7) | 112 (63.3) | 0.73(0.31-1.72) | 0.483 |
| **Ethnicity** |  |  |  |  |
| Relatively advantaged ethnic group* | 18(34.6) | 34 (65.4) | Ref |  |
| Relatively disadvantaged ethnic group * | 58 (38.7) | 92 (61.3) | 1.19 (0.61-2.30) | 0.604 |
| **Family Type** |  |  |  |  |
| Nuclear | 17(25.4) | 50 (74.6) | Ref |  |
| Joint/ Extended | 59(43.7) | 76 (56.3) | 2.28 (1.19-4.36) | **0.012*** |
| **Educational Status of mother** |  |  |  |  |
| Literate | 53(32.1) | 112 (67.9) | Ref |  |
| Illiterate | 23(62.2) | 14 (37.8) | 3.47 (1.65-7.27) | **0.001*** |
| **Educational Status of father** |  |  |  |  |
| Literate | 58(34.5) | 110 (65.5) | Ref |  |
| Illiterate | 18(52.9) | 16 (47.1) | 2.13 (1.01-4.49) | **0.046*** |
| **Mother’s occupation** |  |  |  |  |
| Homemaker | 54(38) | 88 (62) | Ref |  |
| Other than Homemaker | 22(36.7) | 38 (63.3) | 0.94 (0.50-1.76) | 0.855 |
| **Husband’s occupation** |  |  |  |  |
| Agriculture | 3(21.4) | 11(78.6) | Ref |  |
| Non-agriculture | 73(38.8) | 115 (61.2) | 2.32 (0.62- 8.62) | 0.206 |
| **Age of child (in months)** |  |  |  |  |
| Infant (6-12 months) | 33(37.1) | 56 (62.9) | Ref |  |
| Above infant (13 to 23 months) | 43(38.1) | 70 (61.9) | 1.04 (0.58-1.85) | 0.887 |
| **Sex of child** |  |  |  |  |
| Male | 37(34.9) | 69 (65.1) | Ref |  |
| Female | 39(40.6) | 57 (59.4) | 1.27 (0.72-2.25) | 0.402 |
| **Birth weight of child** |  |  |  |  |
| Normal | 62(38) | 101 (62) | Ref |  |
| Low birth | 14(35.9) | 25 (64.1) | 0.91 (0.44-1.88) | 0.804 |
| **Level of knowledge** |  |  |  |  |
| Adequate | 13(29.5) | 31(70.5) | Ref |  |
| Inadequate | 63(39.9) | 95 (60.1) | 1.58 (0.76-3.25) | 0.213 |
| **Parity** |  |  |  |  |
| Primiparous | 28(32.2) | 59 (67.8) | Ref |  |
| Multiparous | 48(41.7) | 67 (58.3) | 1.51 (0.84-2.70) | 0.166 |
| **Mode of delivery** |  |  |  |  |
| Normal | 39(36.4) | 68 (63.6) | Ref |  |
| CS | 37(38.9) | 58 (61.1) | 1.11 (0.62-1.96) | 0.714 |
| **Number of ANC visit** |  |  |  |  |
| <4 times | 14(50.0) | 14(50.0) | Ref |  |
| ≥4 times | 62(35.6) | 112 (64.4) | 0.55 (0.27-2.82) | 0.837 |
| **Number of PNC visit** |  |  |  |  |
| <3 times | 65(41.7) | 91 (58.3) | Ref |  |
| ≥3 times | 11(23.9) | 35 (76.1) | 0.44 (0.21-0.95) | **0.038*** |
| **Breastfeeding problems** |  |  |  |  |
| Yes | 6(10.9) | 49 (89.1) | Ref |  |
| No | 70(47.6) | 77 (52.4) | 7.42 (2.99-18.39) | **<0.001*** |

**Ethnicity was categorized into “advantaged” and “disadvantaged” groups based on Nepal’s social inclusion framework. Brahmin and Chhetri were considered advantaged, whereas Janajati, Madhesi, Muslim, and Dalit were considered disadvantaged groups.*
